# Unveiling the Toxicity of Fine and Nano-Sized Airborne Particles Generated from Industrial Thermal Spraying Processes in Human Alveolar Epithelial Cells

**DOI:** 10.3390/ijms23084278

**Published:** 2022-04-13

**Authors:** Maria João Bessa, Fátima Brandão, Paul H. B. Fokkens, Daan L. A. C. Leseman, A. John F. Boere, Flemming R. Cassee, Apostolos Salmatonidis, Mar Viana, Eliseo Monfort, Sónia Fraga, João Paulo Teixeira

**Affiliations:** 1 Department of Environmental Health, National Institute of Health Dr. Ricardo Jorge, 4000-053 Porto, Portugal; mjbessa8@gmail.com (M.J.B.); fatima.brandao988@gmail.com (F.B.); jpft12@gmail.com (J.P.T.); 2 EPIUnit-Instituto de Saúde Pública, Universidade do Porto, 4050-091 Porto, Portugal; 3 Laboratório para a Investigação Integrativa e Translacional em Saúde Populacional (ITR), 4050-091 Porto, Portugal; 4 Instituto de Ciências Biomédicas Abel Salazar (ICBAS), Universidade do Porto, 4050-313 Porto, Portugal; 5 National Institute for Public Health and Environment (RIVM), 3721 Bilthoven, The Netherlands; paul.fokkens@rivm.nl (P.H.B.F.); daan.leseman@rivm.nl (D.L.A.C.L.); john.boere@rivm.nl (A.J.F.B.); flemming.cassee@rivm.nl (F.R.C.); 6 Institute for Risk Assessment Sciences (IRAS), 3584 Utrecht, The Netherlands; 7 Institute of Environmental Assessment and Water Research, Spanish Research Council (IDAEA-CSIC), 08034 Barcelona, Spain; asalmatonidis@leitat.org (A.S.); mar.viana@idaea.csic.es (M.V.); 8 LEITAT Technological Center, C/de la Innovació 2, 08225 Terrassa, Spain; 9 Institute of Ceramic Technology (ITC), Universitat Jaume I, 12006 Castellón, Spain; eliseo.monfort@itc.uji.es

**Keywords:** A549 cells, cell cycle, cytotoxicity, DNA damage, in vitro toxicity, incidental nanoparticles, H2AX phosphorylation, occupational exposure, process-generated nanoparticles

## Abstract

High-energy industrial processes have been associated with particle release into workplace air that can adversely affect workers’ health. The present study assessed the toxicity of incidental fine (PGFP) and nanoparticles (PGNP) emitted from atmospheric plasma (APS) and high-velocity oxy-fuel (HVOF) thermal spraying. Lactate dehydrogenase (LDH) release, 2-(4-nitrophenyl)-2H-5-tetrazolio]-1,3-benzene disulfonate (WST-1) metabolisation, intracellular reactive oxygen species (ROS) levels, cell cycle changes, histone H2AX phosphorylation (γ-H2AX) and DNA damage were evaluated in human alveolar epithelial cells at 24 h after exposure. Overall, HVOF particles were the most cytotoxic to human alveolar cells, with cell viability half-maximal inhibitory concentration (IC_50_) values of 20.18 µg/cm^2^ and 1.79 µg/cm^2^ for PGFP and PGNP, respectively. Only the highest tested concentration of APS-PGFP caused a slight decrease in cell viability. Particle uptake, cell cycle arrest at S + G_2_/M and γ-H2AX augmentation were observed after exposure to all tested particles. However, higher levels of γ-H2AX were found in cells exposed to APS-derived particles (~16%), while cells exposed to HVOF particles exhibited increased levels of oxidative damage (~17% tail intensity) and ROS (~184%). Accordingly, APS and HVOF particles seem to exert their genotoxic effects by different mechanisms, highlighting that the health risks of these process-generated particles at industrial settings should not be underestimated.

## 1. Introduction

The ceramic industry has been benefitting from nanotechnology innovation processes and advanced materials [1]. Workers from these industries are at risk of exposure to airborne fine (<2.5 µm mass median aerodynamic diameter (MMAD)) and nano-sized (<0.200 µm MMAD) particles that may be released either during the handling or manufacturing of ceramics using engineered nanoparticles (ENP, 1–100 nm) as raw materials or to incidentally emitted particles during mechanical and combustion/heating processes. Indeed, high-energy processes such as laser ablation, laser sintering, physical vapour deposition, inkjet printing, thermal spraying processes (e.g., atmospheric plasma spraying (APS) and high-velocity oxy-fuel spraying (HVOF)) and glazing represent a high potential of fine and ultrafine particle formation and release to the workplace air [2,3,4,5,6,7,8,9,10].

Inhalation is the predominant route of exposure to micro- and nano-sized particles in occupational settings. Respiratory tract deposition and clearance is governed by aerosol physics and by the anatomy and physiology of the respiratory tract [11]. The deeper the particles reach, the harder their removal from the respiratory system, favouring particle–cell interactions that might result in adverse health effects [12,13]. Many epidemiological studies already described an association between exposure to particulate matter (PM) and the occurrence of adverse health effects [14,15]. Exposure to airborne particles has been associated with cardiovascular, pulmonary and neurological diseases, which leads to an increased risk of mortality [16,17,18]. In the case of the ceramic industry, worker exposure to ceramic dusts has been strongly linked to respiratory symptoms, such as wheezing, breathlessness and dry cough, as well as with reduced lung function, chronic bronchitis and chronic obstructive pulmonary disease (COPD) [19,20,21].

Most of the knowledge on nano(particle) toxicity comes from in vitro mechanistic studies. While there are many available studies on the in vitro toxicity of engineered nanoparticles (ENP), little is known about the in vitro hazard of process-generated (nano)particles. In this regard, our group recently conducted a comparative assessment of the in vitro toxicity of ENP (tin oxide (Sb_2_O_3_) and zirconium oxide (ZrO_2_) ENP) that are used as input materials in the ceramic industry vs. particles collected during the HVOF spraying of ceramic coatings onto metal surfaces to produce thermal-resistant coatings [22]. Overall, our data showed that human tri-dimensional (3D) bronchial cultures under air–liquid interface (ALI) conditions were rather resistant to the ENP that induced mild cytotoxicity at early timepoints (24 h), though cells rapidly recovered since no significant changes in cell viability compared to the control were observed at late timepoints (72 h). At the same time, while the fine fraction of the HVOF-derived particles significantly decreased cell viability, the ultrafine fraction significantly increased DNA oxidative damage, showing that HVOF particles exhibited a higher toxicity potential compared to ENP [22]. A recent study by Cediel-Ulloa et al. also evaluated the in vitro toxicity of airborne particles emitted during gas–metal arc welding (GMAW) in a laboratory setting on primary human small airway epithelial cells (hSAEC) [23]. These authors observed that stainless steel welding particles were more cytotoxic compared to mild steel particles and induced oxidative stress in primary human small airway epithelial cells. In addition, Pavlovska et al. investigated the biological effects of airborne particulates collected in woodworking and metalworking industries both on EpiAirway 3D human small airway epithelial cells exposed for 4 h (half a working day), 8 h (full working day) and 72 h (three working days) and on A549 lung epithelial cells that were continuously exposed for 96 h. The obtained data showed that exposure to these polluting particles exerted minor acute effects on the morphology and viability of both A549 cells and EpiAirway tissues. However, a marked reduction in EpiAirway tissue viability after 8 h of exposure to woodworking particles and a slight reduction in tissue viability after 72 h of exposure to metalworking particles were observed [24].

Therefore, the present study aims to further explore the in vitro toxicity of process-generated fine (PGFP; <2.5 µm MMAD) and nano-sized particles (PGNP; <0.200 µm MMAD) that are incidentally emitted during two industrial thermal spraying processes (APS and HVOF) of ceramic coatings onto metal surfaces. We hypothesise that PGNP are more toxic to human alveolar epithelial cells than PGFP. Biological endpoints, including particle internalisation, plasma membrane integrity, cell metabolic activity and viability, reactive oxygen-species (ROS) levels, cell cycle analysis, histone H2AX phosphorylation and DNA damage, were evaluated in human alveolar epithelial A549 cells at 24 h after exposure.

## 2. Results

### 2.1. Process-Generated Fine and Nano-Sized Particle Characterisation

Table 1 presents the physicochemical features of the aqueous suspensions of the tested process-generated particles, namely, concentration (both in terms of mass and number of particles per mL), hydrodynamic size and oxidative potential. As shown, APS-derived aqueous suspensions were more diluted in terms of mass/mL than the HVOF ones, which somehow limited the maximum tested concentrations of the former type of particles, in particular of the PGNP fraction. For APS-derived particles, the mean hydrodynamic size value was 244 nm and 410 nm for PGFP and PGNP, respectively. At the same time, HVOF-PGFP (247 nm) and PGNP (236 nm) exhibited a similar hydrodynamic size mean value. Regarding the oxidative potential, only APS-derived PGFP demonstrated a low oxidative potential, whereas HVOF-derived particles, particularly PGNP, exhibited a high ability to produce •OH in a cell-free environment.

### 2.2. Plasma Membrane Integrity and Cell Viability

**Figure 1:**
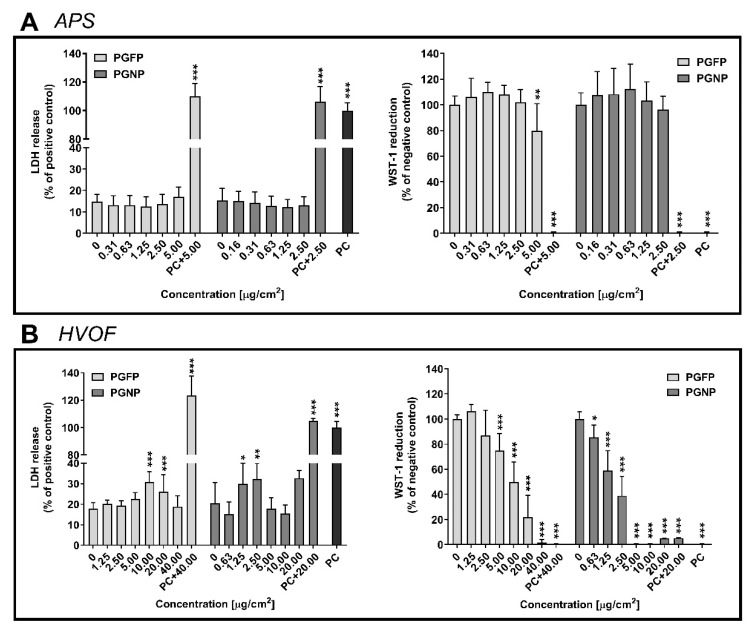
Cytotoxicity of PGFP and PGNP released during APS (**A**) and HVOF (**B**) in human alveolar epithelial cells after 24 h of exposure. Data are expressed as means ± standard deviations (n = 3–4). LDH release values were normalised considering the positive control (total LDH release; cells lysed with 2% Triton X-100), while WST-1 reduction values were normalised considering the negative control (NC). Data were analysed by the one-way analysis of variance (ANOVA) test followed by Dunnett’s post hoc test for multiple comparisons. * *p* ≤ 0.05, ** *p* ≤ 0.01 and *** *p* ≤ 0.001 vs. NC. PC: Positive control (LDH: 2% Triton X-100; WST-1: 70% EtOH).

Under our experimental conditions, no differences in cell membrane integrity and viability were found after 24 h of exposure to both fractions of APS-derived particles compared with the negative control (NC) (Figure 1A). On the other hand, the cytotoxic effects were more pronounced when cells were exposed to particles derived from the HVOF spraying process. While a significant increase in the LDH release compared to control cells was observed after exposure to PGFP at either 10.00 µg/cm^2^ (30.70%) or 20 µg/cm^2^ (26.15%), a clear concentration-dependent decrease in cell viability was detected (Figure 1B). The analysis of cell viability concentration–response curves of cells exposed to HVOF-derived PGFP and PGNP revealed half-maximal inhibitory concentrations (IC_50_) of 20.18 µg/cm^2^ (CI 95%: 11.66–34.95) and 1.79 µg/cm^2^ (CI 95%: 1.48–2.16), respectively.

### 2.3. Intracellular Reactive Oxygen Species Levels

**Figure 2:**
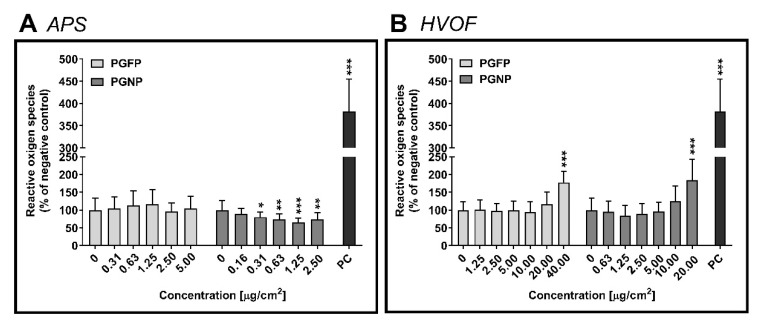
ROS intracellular levels in human alveolar epithelial cells after 24 h of exposure to PGFP and PGNP released during APS (**A**) and HVOF (**B**). Data are expressed as means ± standard deviations (n = 3–4). Values were normalised considering the NC. Data were analysed by the one-way ANOVA test followed by Dunnett’s post hoc test for multiple comparisons. * *p* ≤ 0.05, ** *p* ≤ 0.01 and *** *p* ≤ 0.001 vs. NC. PC: 25 µM AgNO_3_.

No major effects on ROS levels were found on human alveolar epithelial cells after 24 h of exposure to APS-derived PGFP and PGNP (Figure 2A). On the other hand, in cells exposed to the HVOF particles, a significant increase in the intracellular ROS levels was found for the highest tested concentrations of PGFP (176.88 ± 32.35%) and PGNP (183.67 ± 59.06%), when compared to the NC (Figure 2B).

### 2.4. Cellular Uptake of the (Nano)Particles

**Figure 3:**
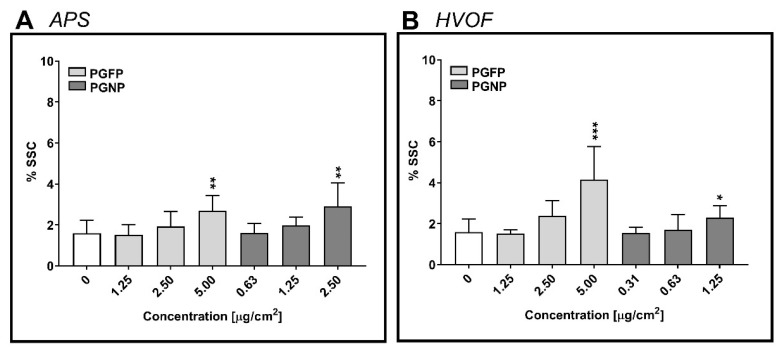
Cellular uptake of PGFP and PGNP released during APS (**A**) and HVOF (**B**) by human alveolar epithelial A549 cells after 24 h of exposure, as estimated by variations in the side scatter signal (SSC). Data are expressed as means ± standard deviations (n = 3–4). Data were analysed by the one-way ANOVA test followed by Dunnett’s post hoc test for multiple comparisons. * *p* ≤ 0.05, ** *p* ≤ 0.01 and *** *p* ≤ 0.001 vs. NC.

The cellular uptake of the process-generated particles under study by human alveolar epithelial cells was estimated based on changes in the percentage of the side scatter signal (%SSC), a measure of cellular complexity, analysed by flow cytometry. As depicted in Figure 3, a concentration-dependent increase in cell complexity was observed in A549 cells that were incubated with all tested particles, regardless of the process and the particle fraction. For APS- and HVOF-derived particles, a significant increase in particle uptake was detected in cells exposed to the highest tested concentrations of either PGFP or PGNP. However, the fine HVOF-derived particles were internalised by A549 cells to a higher degree than the nano-sized fraction. At the same time, HVOF-PGFP (5 µg/cm^2^; 4.16 ± 1.61%) were internalised to a greater extent than APS-PGFP (5 µg/cm^2^; 2.69 ± 0.75%) in A549 cells.

### 2.5. Cell Cycle Analysis

**Figure 4:**
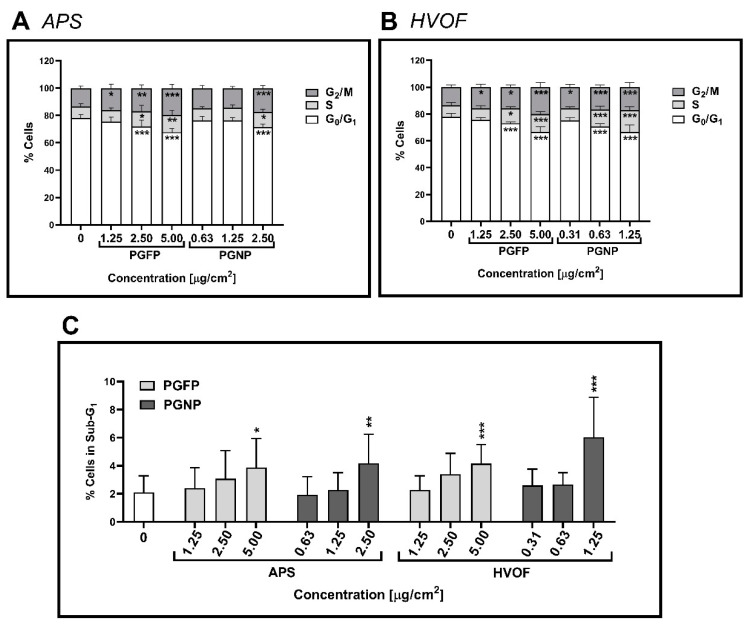
Cell cycle analysis of human alveolar epithelial cells after 24 h of exposure to PGFP and PGNP released during APS (**A**) and HVOF (**B**). The percentage of cells in the sub-G_1_ phase (apoptotic cells) was also analysed (**C**). Data are expressed as means ± standard deviations (n = 3–4). Data were analysed by one-way ANOVA followed by Dunnett’s post-hoc test. * *p* ≤ 0.05, ** *p* ≤ 0.01 and *** *p* ≤ 0.001 vs. NC.

Figure 4 shows the effect of the tested process-generated particles on the human alveolar epithelial cell cycle dynamics, as assessed by flow cytometry. In the NC, most of the cells were at the G_0_/G_1_ phase (78.04 ± 2.51% cells), which was expected, considering that the cells were incubated for 24 h in FBS-free culture medium. Moreover, a concentration-dependent increase in cells in the S and G_2_/M phases was observed at 24 h after exposure to all particles under study, but G_0_/G_1_ cells still represented the largest subpopulation (Figure 4A,B). As shown in Figure 4C, under our experimental conditions, less than 10% of the cells underwent apoptosis (sub-G_1_ population), though a concentration-dependent increase in the apoptotic cell number was observed for all tested particles, regardless of the process and particle fraction.

### 2.6. Histone Gamma-H2AX Phosphorylation

**Figure 5:**
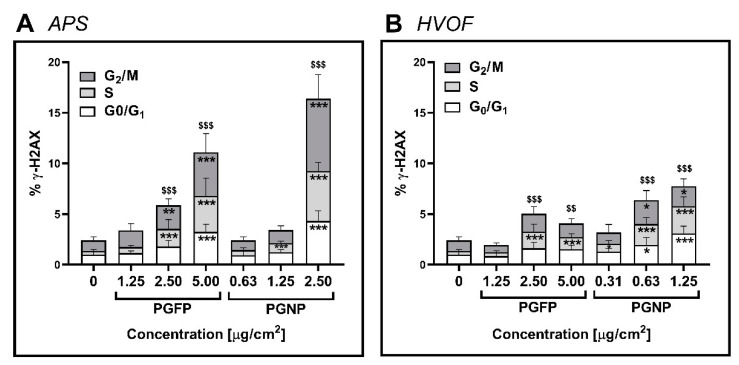
γ-H2AX in human alveolar epithelial cells after 24 h of exposure to PGFP and PGNP released during APS (**A**) and HVOF (**B**). Data are expressed as means ± standard deviations (n = 3–4). Data were analysed by one-way ANOVA followed by Dunnett’s post-hoc test. Global γ-H2AX analysis: ^$$^ *p* ≤ 0.01 and ^$$$^ *p* ≤ 0.001 vs. NC. γ-H2AX in each phase of cell cycle: * *p* ≤ 0.05, ** *p* ≤ 0.01 and *** *p* ≤ 0.001 vs. NC.

Histone gamma-H2AX phosphorylation (γ-H2AX), a biomarker for DNA double-strand breaks was assessed by flow cytometry at 24 h after exposure to the process-generated particles. As shown in Figure 5, a significant increase in the total γ-H2AX levels in A549 cells was detected after exposure to any type of process-generated particles, with this increase being more marked in cells incubated with the APS-derived particles (Figure 5A) compared to the ones exposed to the HVOF particles (Figure 5B). In fact, for the APS-derived particles, PGNP (2.5 µg/cm^2^: 16.39 ± 3.65%) were more effective in causing total γ-H2AX than PGFP (5 µg/cm^2^: 11.10 ± 4.14%). Moreover, increasing levels of γ-H2AX were found for each phase of the cell cycle after 24 h of exposure to the highest tested concentration of PGFP and PGNP APS-derived particles. Regarding HVOF-derived particles, increasing levels of γ-H2AX in each phase of the cell cycle were found but to a much lower degree than APS particles. Camptothecin (3.5 µg/mL) served as a positive control (PC) and, as expected, caused an evident increment in the total γ-H2AX levels in cells at the different cell cycle phases compared to the NC.

**Figure A1:**
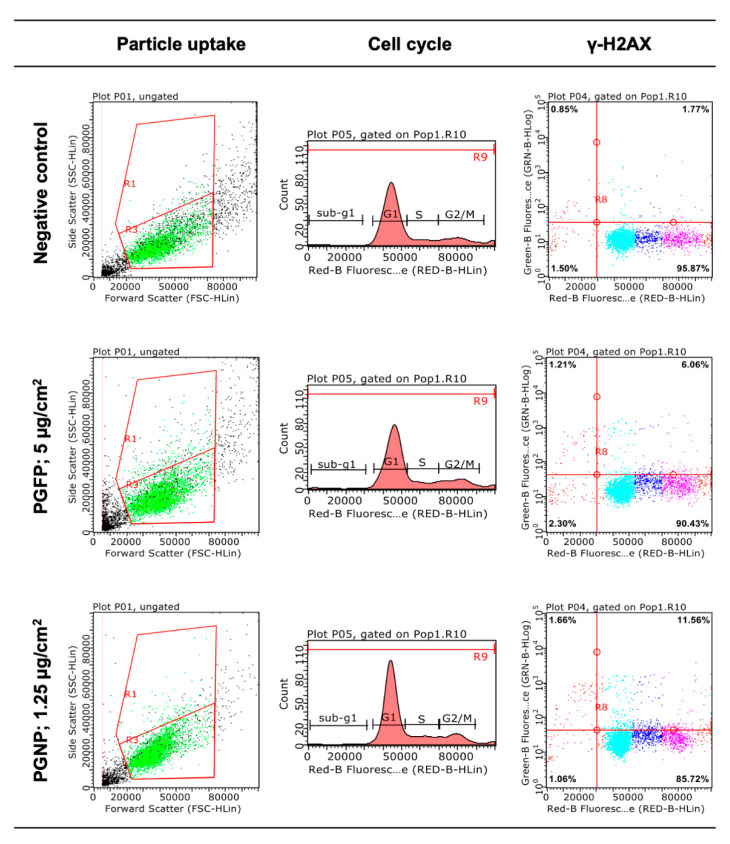
Representative flow cytometry graphs obtained for particle uptake, cell cycle and γ-H2AX analysis in human alveolar epithelial cells incubated for 24 h with the highest tested concentration of process-generated fine (PGFP) and nano-sized (PGNP) particles released during high-velocity oxy-fuel spraying (HVOF).

Representative graphs of the cellular uptake of particles, cell cycle and γ-H2AX analyses by flow cytometry are depicted in Figure A1.

### 2.7. Primary and Oxidative DNA Damage

**Figure 6:**
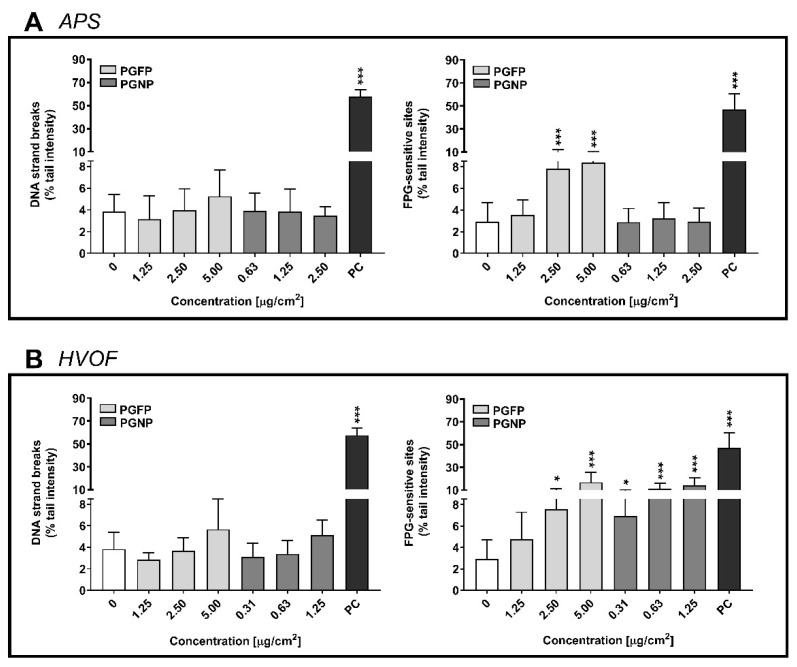
Primary (DNA strand breaks) and oxidative (FPG-sensitive) DNA damage in human alveolar epithelial cells after 24 h of exposure to PGFP and PGNP particles released during APS (**A**) and HVOF (**B**). Data are expressed as means ± standard deviations (n = 3–4). Data were analysed by one-way ANOVA followed by Dunnett’s post-hoc test. * *p* ≤ 0.05and *** *p* ≤ 0.001 vs. NC. PC: 500 µM MMS and 2.5 mM KBrO_3_ for primary and oxidative DNA damage, respectively.

**Figure A2:**
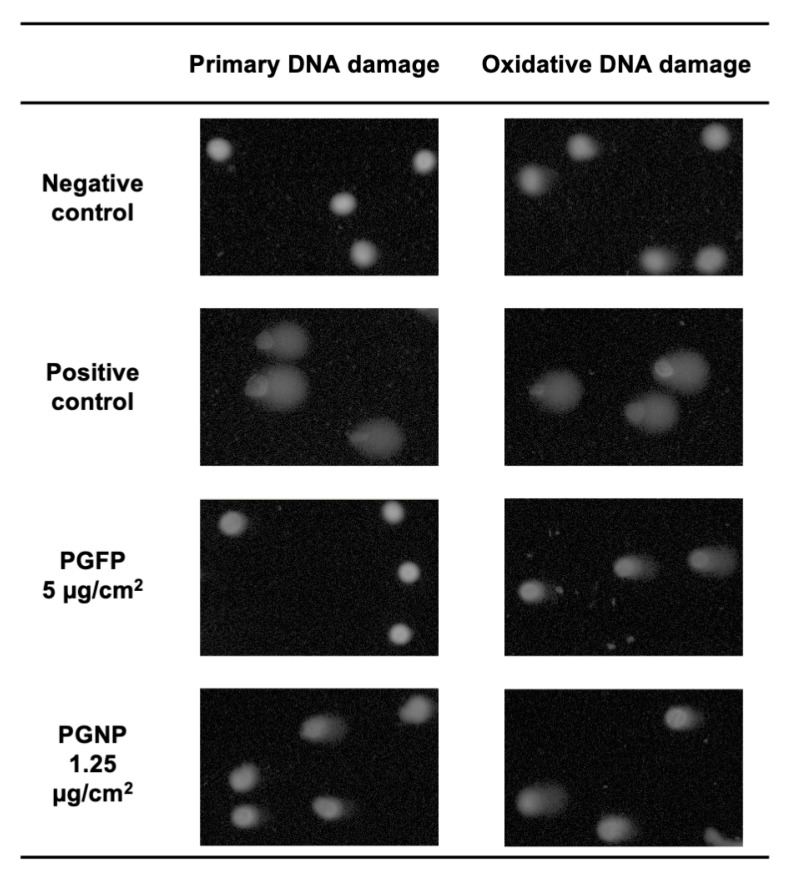
Representative comet assay images (100× magnification) of human alveolar epithelial cells exposed to the highest tested concentration of process-generated fine (PGFP) and nano-sized (PGNP) particles released during high-velocity oxy-fuel spraying (HVOF) and respective experimental controls.

Figure 6 depicts the primary and oxidative DNA damage of A549 cells incubated with the APS- (Figure 6A) and HVOF-derived particles (Figure 6B). Although a slight increase in DNA strand breaks was observed for PGFP of both APS and HVOF spraying processes as well as for HVOF-derived PGNP, those were not significant when compared to the NC. However, APS-derived PGFP significantly increased DNA FPG-sensitive sites of human alveolar epithelial cells at concentrations of 2.5 µg/cm^2^ (7.81 ± 4.40% tail intensity) and 5 µg/cm^2^ (8.37 ± 2.23% tail intensity) compared to the NC (2.90 ± 1.80% tail intensity) (Figure 6A), while the nano-sized fraction did not increase oxidative DNA damage in A549 cells (Figure 6A). HVOF particles seem to cause higher levels of DNA oxidative damage compared to the APS particles. As shown in Figure 6B, both the PGFP and PGNP HVOF-derived fractions significantly increased DNA oxidation in a concentration-dependent manner. Representative comet images of the DNA damage of human alveolar epithelial cells exposed to the highest tested concentrations of PGFP and PGNP emitted during the HVOF thermal spraying process are depicted in Figure A2.

## 3. Discussion

Regardless the size fraction, HVOF-derived particles were more cytotoxic for A549 cultures than the APS particles. Indeed, particles emitted during HVOF spraying induced a marked decrease in cell viability, with PGNP being more potent than PGFP, as evidenced by its ~10× lower IC_50_ value, while only the fine fraction of APS-derived particles slightly decreased cell viability at the highest tested concentration (5 µg/cm^2^). In addition, only exposure to HVOF- but not to APS-emitted particles significantly increased the ROS intracellular levels of A549 cells. These results are in good agreement with the highest oxidative potential of HVOF particles compared to the APS particles. ROS are important molecules that are involved in redox-associated signalling pathways. While important for regulating and maintaining normal physiological functions, excessive levels of intracellular ROS can activate cell death and other signalling pathways, including nuclear factor-κB (NF-κB), mitogen-activated protein kinase (MAPK) and phosphoinositide 3-kinase (PI3-K), that are involved in the regulation of the expression of inflammatory response genes, cell cycle arrest, DNA strand breaks and the formation of 8-oxo-7,8-dihydro-2′-deoxyguanosine (8-oxo-dG) DNA adducts [25,26].

Differences in their chemical composition might have an obvious toxicological impact and may explain the observed cellular effects in A549 cells. The chemical analysis of the airborne fine and nano-sized particles under study revealed a major enrichment in potentially health hazardous metals (chromium (Cr), nickel (Ni) and tungsten (W)) sourcing directly from the feedstock in both scenarios as well as in major elements (aluminium (Al), calcium (Ca) and iron (Fe)) with different possible source origins, including re-suspension of indoor dusts. Size-resolved particle chemical composition analysis by inductively coupled plasma mass spectrometry (ICP-MS) and inductively coupled plasma-optical emission spectrometry (ICP-OES) showed that APS-derived particles were mainly constituted by Al (68% and 42% for PGFP and PGNP, respectively), Cr (11% and 16% for PGFP and PGNP, respectively) and Ni (1% and 21% for PGFP and PGNP, respectively), while the HVOF-generated ones were mainly constituted by Cr (61% and 67% for PGFP and PGNP, respectively) and Ni (27% and 28% for PGFP and PGNP, respectively), as previously reported [2]. These differences in composition might have contributed to the particle aggregation/agglomeration of the aqueous suspensions, in particular of APS-PGNP that presented a high hydrodynamic size value.

Epidemiological and occupational studies have shown the hazard of inhalation exposure to Al, Cr and Ni to human health. Workplace exposure to airborne particles containing these elements has been associated with several respiratory disorders, such as pulmonary fibrosis, asthma, chronic obstructive lung disease, lung cancer and cardiovascular disease [27,28,29,30,31,32,33,34]. Several in vivo and in vitro studies have also addressed the toxicity of the nanoforms of these elements. Kim et al. investigated the toxicity of aluminium oxide (Al_2_O_3_) nanoparticles (NP) following 28 days of repeated exposure by inhalation in male rats and reported a no-observed-adverse-effect level (NOAEL) of 1 mg/m^3^ [35]. At the same time, the existing evidence on the Al_2_O_3_ NP effects on human pulmonary cell lines seems to point out the minimal toxic effects caused by these NP that were considered less toxic when compared to cerium oxide (CeO_2_), titanium dioxide (TiO_2_), silicon dioxide (SiO_2_) and zinc oxide (ZnO) NP [36,37,38,39]. These findings are in line with our results showing that Al-rich APS-derived particles did not induce a marked cytotoxicity or increment in the ROS levels of A549 cells.

Both tested thermal-spraying-derived particles, especially HVOF-emitted ones, are enriched in Cr and Ni. So far, the available in vitro and in vivo studies on Cr and Ni effects have shown pulmonary toxicity in response to exposure to these elements [40,41,42,43,44,45]. In this regard, human lung cells incubated with Cr(VI) have been reported to exhibit significant levels of oxidative DNA damage [46] as well as increasing levels of H2AX-Ser139 phosphorylation [47]. Evidence of cell cycle arrest at G_2_/M has also been found in alveolar A549 cells exposed for 24 h to Cr(VI) [48]. On the other hand, Ding et al. showed that exposure to Ni triggered G_2_/M cell cycle arrest and proliferation blockage in human bronchial epithelial cells (BEAS-2B) [49]. In the nanoscale form, DNA damage accompanied by increased phosphorylation of DNA damage response-associated proteins ATM serine/threonine protein kinase (Ser1981), p53 tumour protein (Ser15) and H2AX (ser139) has been reported by Mo et al. in BEAS-2B cells exposed to Ni NP for 24 h [50].

In response to DNA damage, the cell goes through various checkpoint mechanisms that are essential to survival, but when these fail, rapid cell death is a potential result [51]. DNA damage occurring throughout interphase will elicit a cell cycle arrest that allows time for repair mechanisms to occur prior to progression to the subsequent phases of the cell cycle [52]. For instance, depending on the cell cycle phase at which a double strand break occurs, the repair mechanism used by the cell differs [53]. One mechanism of double strand break repair is through the phosphorylation-dependent recruitment of DNA damage repair factors to sites of DNA damage, such as the phosphorylation of Ser139 on histone H2AX (γ-H2AX) [54]. In the current study, flow cytometry data showed that exposure to any type of thermal-spraying-derived particles induced cell cycle arrest at the S and G_2_/M phases in human alveolar epithelial A549 cells, most likely triggered in response to DNA damage. Indeed, following DNA damage, the S-phase checkpoint delays DNA synthesis, while the G_2_ cell cycle checkpoint prevents cells from entering mitosis, inhibiting cell proliferation [52]. In addition, for every particle, a concentration-dependent increase in the number of apoptotic sub-G_1_ phase cells after 24 h of exposure was observed. Under our experimental conditions, an overall increase in γ-H2AX levels was found in A549 cells, especially in cells at the S and G_2_/M phases. For both types of process-generated particles, PGNP were more effective in increasing γ-H2AX levels compared to PGFP. Interestingly, a more prominent effect on γ-H2AX levels was observed in cells incubated with fine or nano-sized APS-derived particles. On the other hand, although both fractions of HVOF-emitted particles increased the oxidative DNA damage, in cells exposed to PGNP, that effect was visible at lower concentrations. At the same time, oxidative DNA lesions were only observed in cells exposed to the fine fraction of APS-derived particles. Therefore, our data suggest that mechanisms involved in the genotoxicity of the tested thermal-spraying-emitted particles might differ between them. While APS particles caused a marked increase in histone H2AX phosphorylation at serine-139 as an early cellular response to the induction of DNA double-strand breaks, 8-oxo-dG oxidative DNA lesions increased significantly after exposure to the HVOF particles, most likely caused by the increased intracellular ROS levels that were observed.

We have recently reported the effect of both fractions of the HVOF-derived particles studied herein in human 3D bronchial epithelial cultures (MucilAir™) under ALI conditions [22]. We found that PGFP aerosols affected cell viability at dose levels as low as 9 µg/cm^2^, which was not seen for the aerosolised PGNP. However, exposure to PGNP (4.5 mg/cm^2^) caused an increase in the oxidative DNA damage of MucilAir™ cultures. In the present study, under submerged conditions, a stronger toxic response was observed, as both fractions of HVOF particles induced a pronounced decrease in cell viability and higher levels of oxidative DNA damage at lower concentrations after 24 h of exposure in A549 cells. Differences in the magnitude of the responses to the HVOF particles within the two studies may be explained by differences in the attained deposited doses as well as in the sensitivity of the cell models used. While aerosolised particles directly deposit on cell surfaces, in submerged conditions, particles in suspension may react with the culture medium and agglomerate/aggregate into larger particles [55,56]. On the other hand, human primary cultures under ALI conditions have been described as more resistant than the traditional 2D monoculture models since they exhibit a higher degree of complexity, with active ciliary beating and mucus production that mimic the mucociliary clearance defence system that occurs in vivo [57,58], which might have considerably attenuated the uptake and cellular effects of the HVOF-derived particles [22]. Our simplified test system does not account for the differences in pulmonary deposition of PGFP and PGNP, whereas it is well-established that the smaller sizes may penetrate deeper into the lung and more efficiently reach the more vulnerable alveoli. Statements on the actual human health risk are, therefore, not possible in the absence of actual personal exposure levels.

## 4. Materials and Methods

### 4.1. Chemicals

Dimethyl sulfoxide (DMSO), sodium hydroxide (NaOH), sodium chloride (NaCl), potassium chloride (KCl) and potassium hydroxide (KOH) were purchased from Merck KGaA (Darmstadt, Germany). Triton X-100, bovine serum albumin (BSA), low melting point (LMP) agarose, Tris hydrochloride (Tris-HCl), silver nitrate (AgNO_3_), 4-(2-hydroxyethyl)piperazine-1-ethanesulfonic acid (HEPES), methyl methanesulfonate (MMS), propidium iodide (PI), a Roche cytotoxicity detection kit (LDH) and cell proliferation reagent water-soluble tetrazolium (WST-1) were bought from Sigma-Aldrich (Madrid, Spain). Tris-base and disodium salt dihydrate (Na_2_EDTA) were supplied from Merck Millipore (Madrid, Spain). Normal melting point (NMP) agarose was purchased from Bioline (London, UK). Potassium bromate (KBrO_3_) and camptothecin were supplied from Alfa Aesar (Karlsruhe, Germany). Formamidopyrimidine-DNA glycosylase (FPG) was purchased from New England Biolabs (Ipswich, MA, USA). Guava ICF instrument cleaning fluid was supplied by Luminex Corporation (Austin, TX, USA), and pH 7.4 flow cytometry grade PBS from Gibco was purchased from Life Technologies Corp. (Carlsbad, CA, USA). RNAse A from bovine pancreas (DNAse-free) from Applichem Panreac, eBioscience™ phospho-histone H2A.X (Ser139) Alexa Fluor^®^ 488-conjugated monoclonal antibody (CR55T33), Invitrogen™ SYBR^®^ Gold dye and CM-H_2_DCFDA (General Oxidative Stress Indicator) were bought from Thermo Fisher Scientific (Madrid, Spain). All cell culture reagents were purchased from Gibco, Thermo Fisher Scientific (Madrid, Spain). All chemicals used were of high purity or analytical grade.

### 4.2. Fine and Nano-Sized Particle Suspensions and Characterisation

Incidental process-generated fine (PGFP; <2.5 µm MMAD) and nano-sized particles (PGNP; <0.200 µm MMAD) that are emitted during APS and HVOF spraying were collected directly from the inside of the spraying booths at an industrial-scale mechanical workshop in the vicinity of Barcelona, as previously described [2]. Regarding the APS spraying, the collection was performed during the injection of a Cr/Ni (50/50) and Al_2_O_3_ + TiO_2_ feedstock blend, while for HVOF it was performed during the injection of a tungsten carbide (WC)—chromium carbon (CrC)–Ni–cobalt (Co) feedstock blend, as described in [2]. Both the PGFP and PGNP fractions were sampled directly as stock suspensions for toxicity testing, using an aerosol concentration enrichment system, as previously described [59]. The collected samples were subjected to gamma-ray irradiation to ensure the required sterility prior to the cell incubations.

The hydrodynamic size and concentration (number of particles/mL) of the aqueous particle suspensions under study were determined by a nanoparticle tracking analysis (NTA) using a NanoSight LM20 (NANOSIGHT Ltd., Salisbury, UK). The particle oxidative potential (acellular ROS production) was determined by electron spin resonance (ESR) based on the trapping of NP-induced hydroxyl radicals (OH) that are generated in the presence of hydrogen peroxide (H_2_O_2_) using DMPO (5,5-dimethyl-1-pyrroline-N-oxide) as a spin trap, as previously described [60]. Briefly, the particle suspensions were mixed with 0.5 M H_2_O_2_ and 0.05 M DMPO, followed by incubation for 15 min at 37 °C in a heated shaking water bath prior to the ESR analysis. The ESR quantification was conducted with the Analysis Software (2.0 Magnettech GmbH, Berlin, Germany) on first derivation of ESR signals of the DMPOeOH quartet as the average of total amplitudes and was expressed in arbitrary units (A.U.) per sampled volume [60].

### 4.3. Cell Culture

Lung adenocarcinoma epithelial A549 cells from the American Type Culture Collection (ATCC^®^, CCL-185™) were cultured in RPMI 1640 medium with Glutamax™ and 25 mM HEPES and supplemented with 10% heat-inactivated foetal bovine serum (FBS), 50 U/mL penicillin and 50 µg/mL streptomycin. Cells were maintained in a humidified atmosphere with 5% CO_2_ at 37 °C. To carry out the submerged exposure experiments, cells were seeded in 96-well (1.0 × 10^4^ cells/well) or 24-well plates (5.0 × 10^4^ cells/well) and allowed to adhere for 48 h at 37 °C and 5% CO_2_.

### 4.4. Exposure Conditions

All particle stock suspensions under study were dispersed by indirect probe sonication using a Branson sonifier (model 450) equipped with a disruptor cup horn according with the standard operation procedure (SOP) for the preparation of NP suspensions developed within the NanoToxClass project [61]. The selected concentrations of each particle fraction depended on the stock’s concentrations and the volume available. In addition, the tested concentrations were chosen based on the daily alveolar mass dose of 0.13 µg/cm^2^, which was expected to achieve a worst-case occupational exposure scenario with a maximum accumulated lifetime dose of 420 µg/cm^2^, according to Paur et al. [62]. Accordingly, serial dilutions of the stock suspensions were carried out in incubation media (FBS-free cell culture medium), and A549 cells were exposed for 24 h at 37 °C and 5% CO_2_. For APS, the tested particle concentrations ranged from 0.31 to 5.00 µg/cm^2^ and from 0.16 to 2.50 µg/cm^2^ for PGFP and PGNP, respectively, while, for HVOF particles, the concentrations ranged between 2.50 and 40.00 µg/cm^2^ and between 0.63 and 10.00 µg/cm^2^ for PGFP and PGNP, respectively. At least three independent experiments with three replicates each were performed.

### 4.5. Cytotoxicity Assessment

To assess the impact of the tested particles in human alveolar epithelial cells, two endpoints were evaluated: LDH release and WST-1 reduction, indicators of plasma membrane integrity and cell metabolic activity, respectively.

LDH release was determined using a Roche Cytotoxicity Detection Kit (Roche, Mannheim, Germany) according to the manufacturer’s instructions. After exposure and prior to analysis, samples were carefully transferred to 96-well round bottom plates and then centrifuged at 2210× *g* for 5 min to remove the cell debris and residual (nano)particles. Cells lysed with 2% Triton X-100 (30 min) were used as PC. Briefly, 100 µL of freshly prepared reaction mixture was added to 100 µL of each sample and incubated up to 30 min at room temperature and protected from light. The absorbance was measured at 490 nm and 630/690 nm (reference wavelength) in a microplate reader (SpectraMax^®^ iD3 Molecular Devices, San Jose, CA, USA). The LDH release values were normalised considering the PC mean value (total LDH release).

The cell metabolic activity and viability were assessed using the WST-1 Cell Proliferation Reagent Kit (Roche, Mannheim, Germany), according to the manufacturer’s instructions. After exposure and prior to analysis, cells were washed with PBS pH 7.4. Afterwards, 100 µL/well of WST-1 reagent diluted to 1:10 was added for a 2 h incubation period at 37 °C and 5% CO_2_. The sample’s absorbance was measured at 450 nm and 630/690 nm (reference wavelength) in a microplate reader (SpectraMax^®^ iD3 Molecular Devices, San Jose, CA, USA). The WST-1 reduction values were normalised considering the NC mean value.

To test for possible particle interferences with the assays, the PC was determined in the absence and in the presence of the highest tested concentration of particle liquid suspensions. None of tested particles seemed to interfere in the conducted cytotoxicity assays since no significant differences in the PC values in the absence vs. in the presence of the highest tested concentration of PGFP and PGNP were detected.

### 4.6. Intracellular Reactive Oxygen Species Generation

The generation of ROS was estimated using the Invitrogen^TM^ CM-H_2_DCFDA General Oxidative Stress Indicator probe (Thermo Fisher Scientific, Madrid, Spain) according to the manufacturer’s instructions. Briefly, 1.0 × 10^4^ cells/well were seeded in 96-well black clear-bottom plates and the medium was renewed after 24 h. At 48 h post-seeding, cells were loaded with 5 µM CM-H_2_DCFDA probe for 1 h at 37 °C and 5% CO_2_. Then, the medium was aspirated, and cells were exposed to the tested particles over a 24 h period. Following exposure, the fluorescence was measured at 492 nm/527 nm (excitation/emission) in a microplate reader (SpectraMax^®^ iD3 Molecular Devices, San Jose, CA, USA). The ROS production was normalised considering the mean fluorescence (arbitrary units) of the NC.

### 4.7. Cellular Uptake, Cell Cycle and Histone Gamma-H2AX Phosphorylation Analysis by Flow Cytometry

The determination of cellular particle uptake, changes in the cell cycle by determining cellular distribution in the different phases (G_0_/G_1_, S, G_2_/M and Sub-G_1_) and DNA double-strand breaks assessed via phosphorylation of the Ser-139 residue of the histone variant H2AX (γ-H2AX) were carried out by flow cytometry using a Guava^®^ easyCyte™ flow cytometer (Merck KGaA, Darmstadt, Germany). Briefly, 5.0 × 10^4^ cells/well were seeded onto 24-well plates, with medium renewal after 24 h. At 48 h post-seeding, cells were exposed to three non-cytotoxic concentrations of each particle over a 24 h period, followed by medium removal and the washing of the cells with pH 7.4 PBS. Cells were detached using 0.05% trypsin-EDTA, inactivated with incubation medium and centrifuged at 900× *g* for 5 min. The supernatant was gently removed, and the cells were permeabilised and fixed with ice-cold 70% ethanol and left overnight at −20 °C. To remove the ethanol, the samples were centrifuged, washed with PBS with 1% BSA and once again centrifuged at 900× *g* for 5 min. Then, the cells were labelled with 5 µg/mL Phospho-Histone H2A.X (Ser139) Alexa Fluor^®^ 488-conjugated monoclonal antibody for 15 min at room temperature and protected from light, followed by a washing step with PBS with 1% BSA and centrifugation at 900× *g* for 5 min. Prior to analysis, a final 15 min staining at room temperature with a 50 µg/mL RNAse and 50 µg/mL PI solution was performed to ensure that only nuclear DNA was stained. Acquisitions were made with approximately 5000 events/per sample and recorded at a low flow rate (0.24 μL/s). For estimating the potential of particles to enter cells, the analysis was carried out by measuring the size (forward scatter, FSC) and complexity (side scatter, SSC) of the cells, following the protocol described by Suzuki, et al. [63]. Debris and doublets were gated out by plotting SSC-width vs SSC-area. A cell cycle analysis was performed by evaluating the relative cellular DNA content from the PI signal detection, as previously described by Rosário, et al. [64], while γ-H2AX was measured by assessing the Alexa Fluor^®^ 488 and PI channel intensities, as described by Valdiglesias, et al. [65]. Camptothecin at 3.5 µg/mL was used as a PC to help define where cells were positive for γ-H2AX. The data were analysed using the Guava^®^ InCyte^TM^ Software (Merck KGaA, Darmstadt, Germany).

### 4.8. DNA Damage Assessment

Primary and oxidative DNA damage were assessed by the standard alkaline and FPG-modified comet assay versions, respectively, as previously described [60]. The Minimum Information for Reporting Comet Assay procedures and results (MIRCA) recommendations were followed in this manuscript [66]. Briefly, 5.0 × 10^4^ cells/well were seeded onto 24-well plates, with medium renewal after 24 h. At 48 h post-seeding, cells were exposed for 24 h to three non-cytotoxic concentrations of each tested particle. After exposure, cells were washed twice with PBS pH 7.4, scrapped and suspended in PBS pH 7.4. Cells exposed to 500 µM MMS and 2.5 mM of KBrO_3_ for 30 min were included as a PC for primary and oxidative DNA damage assessment, respectively. The cells were counted in a Neubauer’s chamber and 6.0 × 10^3^ cells were transferred to a microcentrifuge tube, centrifuged at 700× *g* for 5 min and then embedded in 100 μL of 1% LMP agarose. Five μL of each sample was placed on microscope slides precoated with 1% NMP using a high-throughput system of a 12-gel comet assay unit (Severn Biotech Ltd.^®^, Kidderminster, UK) and placed for 5 min at 4 °C. Duplicates of each sample were added per slide. Slides were performed in triplicate, one for the alkaline version and the other two for the enzymatic version of the comet assay (with or without the FPG enzyme). Then, slides were immersed in an ice-cold lysis solution (NaCl 2.5 M, Na_2_EDTA 100 mM, Tris-base 10 mM, NaOH 10 M, pH 10, Triton-X 100 1%) for 2 h at 4 °C. For the enzymatic version, the slides were washed in freshly prepared ice-cold buffer F solution (HEPES 400 mM, KCl 1 M, Na_2_EDTA 5 mM, BSA 2 mg/mL, pH 8.0) (3 × 5 min) at 4 °C. A 30 μL solution of buffer F or FPG enzyme (2.7 U/mL) was added to each well and incubated for 30 min at 4 °C. For this to happen, slides were previously placed on an ice-cold metal base of a 12-gel chamber apparatus (Severn Biotech Ltd.^®^, Kidderminster, UK) covered by a silicon rubber 12-well mould, followed by a top plate and clamped. Meanwhile, slides for the alkaline version remained in lysis solution. All slides were immersed in electrophoresis solution (Na_2_EDTA 1 mM, and NaOH 0.3 M, pH 13) in the electrophoresis platform for 40 min, followed by electrophoresis for 30 min at a constant 25 V (0.9 V/cm) and 400 mA. For slide washing, these were first covered by cold PBS (pH 7.2) and then by deionised H_2_O for 10 min each. At the end of electrophoresis, slides were neutralised and fixed as described elsewhere [67]. For comet scoring, slides were initially hydrated in TE buffer (Tris-HCl 10 mM and EDTA 1 mM, pH 7.5–8) and stained at room temperature with a 1:10,000 dilution of SYBR^®^ Gold in TE buffer for 40 min. Comets were visualised using a Motic BA410 ELITE Series microscope equipped with a Complete EPI-Fluorescence Kit and scored using the Comet Assay IV image analysis software (Perceptive Instruments, Staffordshire, UK). At least 100 cells/experimental group (50 in each replicate gel) were scored. The comet tail DNA percentage (% tail intensity) was used as a DNA damage descriptor.

### 4.9. Statistical Analysis

A statistical analysis was performed using SPSS (version 26.0, Armonk, NY, USA) and GraphPad Prism (version 6.0, San Diego, CA, USA) statistical software. The experimental data were expressed as means ± standard deviations (SD). The data were tested for normality and homogeneity of variances by the Shapiro–Wilk and Levene’s tests, respectively. For each assessed timepoint, the differences between the tested concentrations and NC were estimated using a one-way ANOVA followed by a post-hoc Dunnett’s test for multiple comparisons. A *p* value < 0.05 was considered significant.

## 5. Conclusions

HVOF particles were more cytotoxic compared to APS particles, most likely due to differences in their elemental composition. As hypothesised, PGNP derived from HVOF were more cytotoxic to A549 cells than PGFP, while both fractions of APS-emitted particles did not induce significant cytotoxic effects in A549 cells. Notwithstanding, particles emitted from the two thermal spraying processes under study were genotoxic to human alveolar epithelial cells. While APS particles markedly increased the levels of H2AX phosphorylation, HVOF particles caused 8-oxo-dG oxidative DNA lesions, and the effects were evident at lower concentrations of PGNP compared to PGFP.

Our data highlight that workers from industries employing high-energy processes may be at (increased) risk of adverse health effects depending on the actual inhaled dose, i.e., exposure levels and duration. Occupational epidemiological studies are urgently needed to establish this risk, whereas a better understanding of the cellular mechanisms involved in process-generated particle-induced biological effects, ultimately contributing to controlling exposures to these particles in the workplace, would facilitate reducing the health risks. The availability of information obtained from real-world exposure scenarios is deemed essential to establishing realistic preventive and corrective measures adapted to the different work scenarios (manufacturing technologies and/or chemical composition of materials).

## Figures and Tables

**Table 1:** Physicochemical characteristics of the tested PGFP and PGNP aqueous suspensions.

		Stock Suspension Concentration (mg/mL)	Stock Suspension Concentration (Number of Particles/mL)	Hydrodynamic Size (nm)	Oxidative Potential (A.U.) *
APS	PGFP	0.068	8.49 × 10^8^	244 ± 120	3291
	PGNP	0.034	4.21 × 10^8^	410 ± 162	5319
HVOF	PGFP	1.069	9.72 × 10^8^	247 ± 116	9893
	PGNP	0.140	15.86 × 10^8^	236 ± 86	12833

Data are presented as means ± SD. Hydrodynamic size and concentration were measured by nanoparticle tracking analysis. Oxidative potential was measured by electronic spin resonance. A.U.: arbitrary units. * Negative control (ultrapure water) = 3191 A.U.; Positive control (DOFA) = 48,041 A.U.
